# Systemic gene therapy with thymosin β4 alleviates glomerular injury in mice

**DOI:** 10.1038/s41598-022-16287-z

**Published:** 2022-07-16

**Authors:** William J. Mason, Daniyal J. Jafree, Gideon Pomeranz, Maria Kolatsi-Joannou, Antje K. Rottner, Sabrina Pacheco, Dale A. Moulding, Anja Wolf, Christian Kupatt, Claire Peppiatt-Wildman, Eugenia Papakrivopoulou, Paul R. Riley, David A. Long, Elisavet Vasilopoulou

**Affiliations:** 1 Division of Natural Sciences, Medway School of Pharmacy, University of Kent, Chatham, Kent UK; 2 Developmental Biology and Cancer Programme, UCL Great Ormond Street Institute of Child Health, London, UK; 3 UCL MB/PhD Programme, Faculty of Medical Science, University College London, London, UK; 4 Medizinische Klinik und Poliklinik I, University Clinic Rechts der Isar, TUM Munich, Munich, Germany; 5 DZHK (German Center for Cardiovascular Research), Partner Site Munich Heart Alliance, Munich, Germany; 6 Division of Natural Sciences, University of Kent, Chatham, Kent UK; 7 Department of Internal Medicine and Nephrology, Clinique Saint Jean, Brussels, Belgium; 8 Department of Physiology, Anatomy and Genetics, University of Oxford, Oxford, UK; 9 Comparative Biomedical Sciences, The Royal Veterinary College, Royal College Street, London, NW1 0TU UK

**Keywords:** Cell biology, Nephrology, Kidney

## Abstract

Plasma ultrafiltration in the kidney occurs across glomerular capillaries, which are surrounded by epithelial cells called podocytes. Podocytes have a unique shape maintained by a complex cytoskeleton, which becomes disrupted in glomerular disease resulting in defective filtration and albuminuria. Lack of endogenous thymosin β4 (TB4), an actin sequestering peptide, exacerbates glomerular injury and disrupts the organisation of the podocyte actin cytoskeleton, however, the potential of exogenous TB4 therapy to improve podocyte injury is unknown. Here, we have used Adriamycin (ADR), a toxin which injures podocytes and damages the glomerular filtration barrier leading to albuminuria in mice. Through interrogating single-cell RNA-sequencing data of isolated glomeruli we demonstrate that ADR injury results in reduced levels of podocyte TB4*.* Administration of an adeno-associated viral vector encoding TB4 increased the circulating level of TB4 and prevented ADR-induced podocyte loss and albuminuria. ADR injury was associated with disorganisation of the podocyte actin cytoskeleton in vitro, which was ameliorated by treatment with exogenous TB4. Collectively, we propose that systemic gene therapy with TB4 prevents podocyte injury and maintains glomerular filtration via protection of the podocyte cytoskeleton thus presenting a novel treatment strategy for glomerular disease.

## Introduction

One in ten people worldwide has chronic kidney disease (CKD)^1^. A subset of patients progresses to end-stage kidney disease (ESKD), which requires dialysis or transplantation and is a risk factor for cardiovascular disease and all-cause mortality^1^. CKD progression is linked to breakdown of the glomerular filtration barrier, the site of ultrafiltration in the kidney, which consists of endothelial cells, the glomerular basement membrane (GBM) and epithelial podocytes^2,3^. Podocytes have a unique architecture with foot processes that extend from their cell bodies, interdigitate and form slit diaphragms facilitating size and charge-selective filtration and preventing the loss of plasma proteins^4,5^. In health, podocyte shape is maintained by a complex, highly regulated actin cytoskeleton, which supports the foot processes^6,7^, and anchors the cell to the GBM^8^. During glomerular disease, the podocyte cytoskeleton becomes disorganised often leading to podocyte loss, impaired filtration and leakage of plasma proteins, such as albumin, into the urine^7,9–11^. Albuminuria is a hallmark of glomerular disease, irrespective of the underlying aetiology^12^. Therefore, therapies that protect the podocyte cytoskeleton represent a novel strategy to preserve the integrity of the glomerular filtration barrier, prevent albuminuria and improve glomerular disease progression.

Thymosin β4 (TB4) sequesters monomeric G-actin in mammalian cells^13,14^ and maintains high concentrations of G-actin available for polymerisation into actin filaments (F-actin)^15^. We have previously shown that endogenous TB4 is expressed in podocytes and has a protective role. We found that lack of endogenous TB4 worsens albuminuria, renal function and glomerular injury in a mouse model of immune-mediated glomerular disease, concomitant with loss of podocytes from the glomerular filtration barrier. Furthermore, a direct role for endogenous TB4 on the podocyte cytoskeleton was established in vitro, where loss of TB4 resulted in a shift from cortical actin to cytoplasmic actin stress fibers and enhanced podocyte migration^16^.

These findings raise the possibility that treatment with exogenous TB4 could be used therapeutically to protect the podocyte cytoskeleton and slow glomerular disease progression. TB4 has already shown promise as a treatment for a diverse range of conditions, such as myocardial infarction^17^, dry eye syndrome^18^, stroke^19^ and inflammatory lung disease^20^. Furthermore, exogenous TB4 is protective in animal models of kidney injury^21^, including diabetic nephropathy^22^, unilateral ureteral obstruction^23,24^ and acute ischaemia reperfusion injury^25^, however none of these studies examined the specific effect of exogenous TB4 on podocytes. Additionally, the studies above administered TB4 peptide, which has a relatively short half-life with enhanced levels in the plasma evident for only 6 h following injection^26^. To overcome the rapid metabolic turnover of TB4, recombinant adeno-associated virus (AAV)-mediated gene therapy can be utilised to induce stable, long term transgene expression via a single injection^27–29^. Indeed, TB4-encoding AAV constructs have been successfully used for tissue-specific (muscle, heart) or systemic upregulation of TB4 in mouse, rabbit and pig disease models with therapeutic effects^30–32^.

Here, we show that podocyte injury induced by Adriamycin (ADR) is associated with reduced expression of endogenous TB4 in podocytes. Using systemic gene therapy, upregulation of the plasma concentration of TB4 was able to prevent ADR-induced albuminuria and podocyte loss. Further examination of the podocyte F-actin structures in vitro revealed that exogenous TB4 prevented ADR-induced cytoskeletal disorganisation. Collectively, our work shows that gene therapy-mediated systemic administration of TB4 presents a novel treatment strategy to protect podocytes from injury and preserve the integrity of the glomerular filtration barrier.

## Results

### ADR is associated with the downregulation of TB4 in podocytes

**Figure 1:**
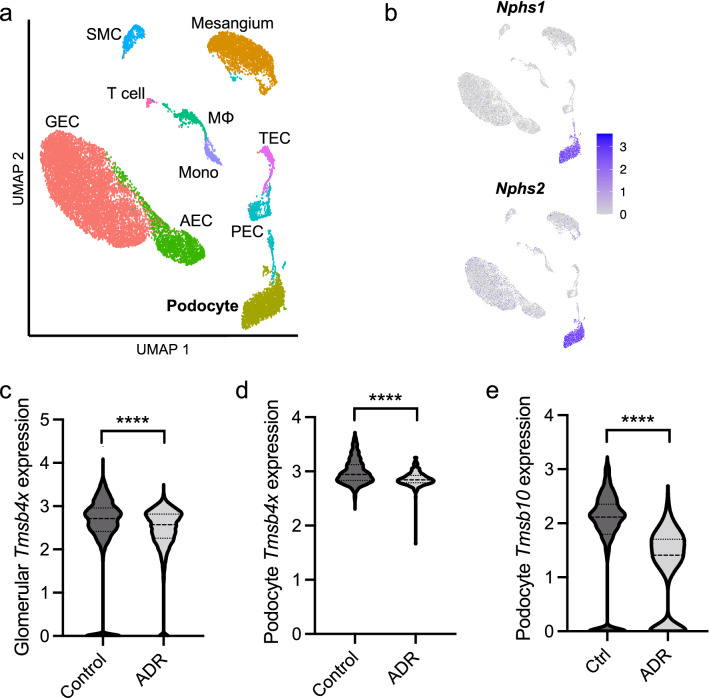
Podocyte-specific *Tmsb4x* and *Tmsb10* expression is downregulated in murine Adriamycin nephropathy. **(a)** Uniform manifold approximation and projection (UMAP) from single-cell RNA sequencing data of 8412 glomerular cells from two wildtype (control) mice and 8296 glomerular cells from mice with Adriamycin nephropathy (ADR). After analysis and cell type assignment, ten transcriptionally distinct cell populations were discriminated including arterial endothelial cells (AEC), glomerular endothelial cells (GEC), macrophages (MΦ), mesangial cells, monocytes (Mono), parietal epithelial cells (PEC), podocytes, smooth muscle cells (SMC), T cells, tubular epithelial cells (TEC). The markers used for cell type identification and assignment, and the numbers of cell types per condition, are shown in Supplementary Fig. 1. **(b)** Feature plots showing expression of nephrin *(Nphs1)* and podocin (*Nphs2),* canonical markers of podocytes, across the dataset. **(c)** Violin plot comparing the scaled expression of *Tmsb4x* of all glomerular cells (podocytes, GECs, mesangium) between ADR (*n* = 5602 cells) and control (*n* = 7190 cells). An average log fold decrease of 0.23 was detected in ADR compared to control (*: adjusted *P* value < 0.0001). **(d)** Violin plot comparing the scaled expression of *Tmsb4x* of podocytes between ADR (*n* = 378 cells) and control (*n* = 1486 cells). An average log fold decrease of 0.52 was detected in ADR compared to control (****: adjusted *P* value < 0.0001). **(e)** Violin plot comparing the scaled expression of *Tmsb10* of podocytes between ADR and control. An average log fold decrease of 1.52 was detected in ADR compared to control (****: adjusted *P* value < 0.0001).

ADR, a chemotherapeutic drug, replicates some of the features of human focal segmental glomerulosclerosis (FSGS)^33^ and has toxic effects on podocytes in vitro and in vivo, including cytoskeletal disorganisation, foot process effacement and loss of podocyte viability^10,34,35^. Firstly, we examined if ADR administration altered podocyte TB4 expression in mice. To assess the expression of *Tmsb4x*, the gene encoding the TB4 protein, specifically in podocytes, we analysed a published scRNAseq dataset obtained from glomeruli of healthy or ADR-injured C57BL/6J mice^36^. Using unsupervised clustering analysis we derived ten transcriptionally distinct cell types (Fig. 1a), which using established markers of glomerular cell types, we identified to represent all the known glomerular cell types (Supplementary Fig. 1a). Confirming the identity of podocytes, the podocyte cluster expressed both nephrin (*Nphs1*) and podocin (*Nphs2*), components of the podocyte slit diaphragm (Fig. 1b). Comparison of control and ADR-injured glomeruli showed representation of all cell types in both groups (Supplementary Fig. 1b). As previously described^36^, we found a reduction in the proportion of podocyte cells in the ADR-injured glomeruli compared with controls (Supplementary Fig. 1c). ADR injury was associated with significant downregulation of *Tmsb4x* in the glomerular tuft, assessed by grouped analysis of glomerular endothelial, mesangial and podocyte cells (Fig. 1c; 0.23 log fold change; *P* < 0.0001) and a more pronounced, 0.52 log fold reduction, in podocytes (Fig. 1d; *P* < 0.0001). The expression of *Tmsb10*, another member of the β thymosins family, which is enriched in podocytes^37^, was also reduced in podocytes (Fig. 1e; 1.52 log fold change; *P* < 0.0001). To assess whether the downregulation of *Tmsb4x* and *Tmsb10* mRNA in podocytes is also evident in other mouse models of glomerular disease, we analysed the scRNAseq datasets obtained from glomeruli of mice with nephrotoxic nephritis or BTBR *ob/ob* (*Lepr*^*Ob/Ob*^) mice with diabetic kidney disease^36^. The podocyte expression of both *Tmsb4x* and *Tmsb10* was reduced in both mice with nephrotoxic nephritis or diabetes compared with healthy animals (Supplementary Fig. 2; *P* < 0.0001).

### Systemic upregulation of TB4 using gene therapy

**Figure 2:**
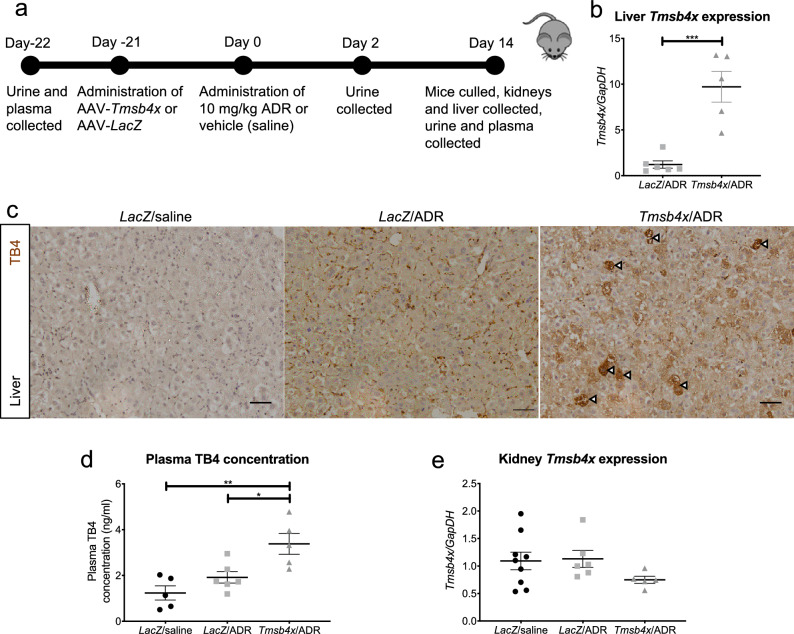
AAV2/7 causes systemic upregulation of thymosin β4 in vivo. (**a**) BALB/c mice were injected with AAV-*LacZ* or AAV-*Tmsb4x* three weeks prior to 10 mg/kg ADR or vehicle (saline) administration and culled 14 days after ADR/saline injection at which point thymosin β4 levels were quantified. (**b**) Liver expression of *Tmsb4x* mRNA (unpaired *t* test)*.* (**c**) Expression of TB4 peptide in the liver. Arrowheads refer to cells with particularly high expression of TB4. Scale bars = 50 µm. (**d**) Plasma concentration of TB4 (one-way ANOVA with Tukey post hoc test). (**e**) Whole kidney expression of *Tmsb4x* gene (one-way ANOVA with Tukey post hoc test). *LacZ*/saline, *n* = 9; *LacZ*/ADR, *n* = 6; *Tmsb4x*/ADR, *n* = 5. Data are presented as the mean ± SEM; *P ≤ 0.05, **P ≤ 0.01 and ***P ≤ 0.001. TB4, *Tmsb4x,* thymosin β4; ADR, Adriamycin; *LacZ*, β-Galactosidase; AAV, adeno-associated virus.

We then assessed the effects of exogenous TB4 administration on ADR-induced glomerular disease. We took a preventative strategy and BALB/c mice were administered intravenously AAV-*Tmsb4x* or AAV-*LacZ* as a control^30,32^. Three weeks after AAV injection, ADR was administered (day 0). Fourteen days later the animals were euthanised and we compared mice administered either: (i) AAV-*Tmsb4x* and ADR *(Tmsb4x*/ADR); (ii) AAV-*LacZ* and ADR (*LacZ*/ADR) and (iii) AAV-*LacZ* and saline as our control group (*LacZ*/saline) (Fig. 2a). Initially, we examined if AAV-*Tmsb4x* altered *Tmsb4x* mRNA levels in the liver, the primary site of transduction following systemic AAV 2/7 administration^38^. We found a tenfold increase in liver *Tmsb4x* mRNA levels in the *Tmsb4x*/ADR group compared with the *LacZ*/ADR group (*P* = 0.0004) (Fig. 2b). This corresponded with strong positive immunostaining for TB4 in the liver in *Tmsb4x*/ADR mice (Fig. 2c). Additionally, as TB4 is a secreted peptide^39^, we measured circulating levels and found a twofold increase in the *Tmsb4x/*ADR group compared with either the *LacZ*/ADR (*P* = 0.0217) or *LacZ*/saline group (*P* = 0.0021) (Fig. 2d). Using qRT-PCR on whole kidney lysates we found no difference in *Tmsb4x* mRNA levels between the three experimental groups (Fig. 2e) in agreement with previous reports that AAV 2/7 does not target the kidney^38^ . To assess whether circulating TB4 peptide can reach the podocytes, we injected *Tmsb4x* knockout mice lacking endogenous TB4 protein with AAV-*Tmsb4x* or AAV-*LacZ*. Positive immunostaining for TB4 in podocytes co-labelled with synaptopodin was detected in *Tmsb4x* knockout mice injected with AAV-*Tmsb4x* but not in mice injected with AAV-*LacZ* (Supplementary Fig. 3).

### TB4 prevents ADR-induced albuminuria

**Figure 3:**
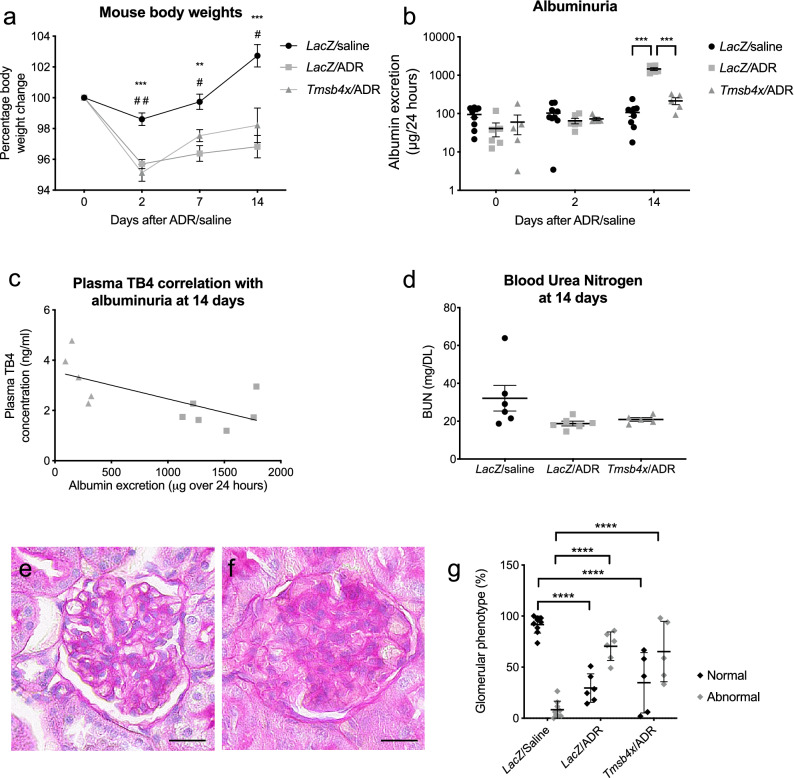
Renal function following ADR/TB4 treatment. **(a)** Mouse weight change 2, 7 and 14 days after ADR/vehicle injection. Statistical annotations (two way repeated measures ANOVA with Tukey post hoc test) using asterisk refer to comparison between *LacZ*/saline & *LacZ*/ADR. Annotations using hash refer to comparison between *LacZ*/saline & *Tmsb4x*/ADR. **(b)** Twenty-four hour urinary albumin excretion 0, 2 and 14 days after ADR/vehicle injection (two way repeated measures ANOVA with Tukey post hoc test). **(c)** Correlation of plasma TB4 concentration with urinary albumin 14 days after ADR injection (Linear regression, *R*^2^ = 0.4584, *P* ≤ 0.05). **(d)** Blood urea nitrogen concentration 14 days after ADR/vehicle injection (one-way ANOVA with Tukey post hoc test). Representative images of normal **(e)** and abnormal **(f)** glomeruli used to assess glomerular morphology, scale bar = 20 μm. **(g)** Glomerular phenotype 14 days after ADR/vehicle injection. Results for each category are expressed as a percentage of the total glomeruli assessed. *LacZ*/saline, *n* = 9; *LacZ*/ADR, *n* = 6; *Tmsb4x*/ADR, *n* = 5. Data are presented as the mean ± SEM; *P ≤ 0.05, **P ≤ 0.01, ***P ≤ 0.001 and ****P ≤ 0.0001. TB4, *Tmsb4x,* thymosin β4; ADR, Adriamycin; *LacZ*, β-galactosidase.

Next, we examined the effect of TB4 administration on ADR injury in vivo. Firstly, as an overall measure of health, we weighed the mice throughout the time course of the experiment. All mice administered ADR lost a significantly greater proportion of their body weight 48 h following ADR injection compared with *LacZ*/saline animals (Fig. 3a). This difference was sustained at both day 7 and 14 after ADR injection. At all time-points there was no difference between ADR mice administered AAV-*LacZ* and AAV-*Tmsb4x*.

We then analysed the levels of albumin in the urine (μg/24 h), a marker of glomerular filtration barrier integrity^40^. Prior to ADR injection, there was no difference in the levels of urinary albumin (mean ± SEM of *LacZ*/saline group: 95 ± 16, *LacZ*/ADR group: 41 ± 16, *Tmsb4x*/ADR group: 60 ± 32). Similar levels were found two days after ADR administration with no differences between the three groups of mice (*LacZ*/saline group: 103 ± 20, *LacZ*/ADR group: 65 ± 11, *Tmsb4x*/ADR group: 73 ± 7). Conversely, at 14 days post ADR injection, albuminuria significantly increased to 1448 ± 115 in the *LacZ*/ADR mice compared with 107 ± 22 in *LacZ*/saline animals (*P* = 0.001). Strikingly, gene therapy with TB4 prevented this increase with a urinary albumin excretion of 214 ± 43 in the *Tmsb4x*/ADR group (*P* < 0.0001 versus *LacZ*/ADR group) (Fig. 3b). We analysed the relationship between plasma TB4 concentration and albuminuria in *LacZ*/ADR and *Tmsb4x*/ADR mice 14 days post ADR injection and found a negative correlation (*R*^*2*^ = 0.4584, *P* = 0.0221, Fig. 3c). Urea is freely filtered through the glomerular filtration barrier and increased plasma blood urea nitrogen (BUN) concentration is an indicator of declining kidney function^41^. However, we found that there were no significant changes between any of the groups 14 days after ADR administration (Fig. 3d). Assessment of gross glomerular morphology revealed that both *LacZ*/ADR and *Tmsb4x*/ADR mice had significantly more abnormal glomeruli compared with *LacZ*/saline mice (*P* < 0.0001), but there was no significant decrease between *LacZ*/ADR and *Tmsb4x*/ADR mice (Fig. 3e–g).

Previously the protective effect of TB4 in CKD has been linked to its anti-inflammatory properties^16,24,42^. We quantified F4/80^+^ macrophage numbers within and around the glomerular tuft (Supplementary Fig. 4a). Within the glomerular tuft, macrophages were rare across all three groups (Supplementary Fig. 4b). The number of macrophages outside the glomerular tuft was similar among the groups (Supplementary Fig. 4c), excluding a role for macrophage accumulation in our model and suggesting that the protective effect of TB4 in ADR-induced glomerular injury occurs by other means.

### TB4 prevents ADR-induced podocyte injury in vivo

**Figure 4:**
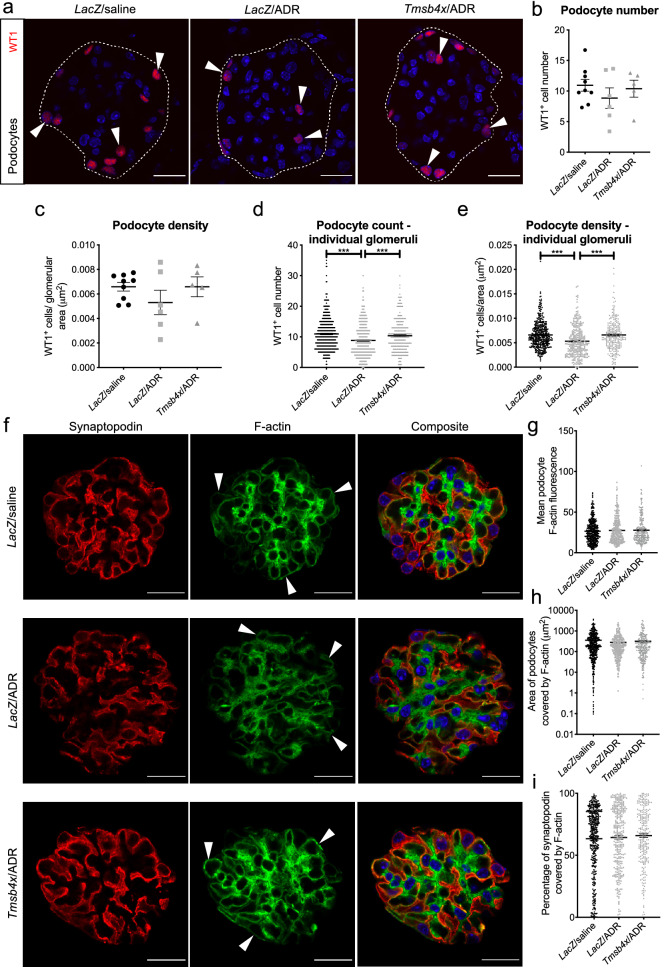
Analysis of podocytes in vivo after ADR/TB4 treatment. **(a)** Representative images of WT1^+^ cells in glomeruli from *LacZ*/saline, *LacZ*/ADR and *Tmsb4x*/ADR treated mice. White arrowheads indicate podocyte nuclei. White dashed line indicates glomerular tuft boundary. Quantification of **(b)** number of WT1^+^ cells in glomeruli and **(c)** glomerular WT1^+^ density. Individual data points represent average values per mouse (*LacZ*/saline, *n* = 9; *LacZ*/ADR, *n* = 6; *Tmsb4x*/ADR, *n* = 5 mice; one-way ANOVA with Tukey post hoc test) and 50 glomeruli were assessed per mouse. Quantification of **(d)** WT1^+^ cell count and **(e)** glomerular WT1^+^ density with each data point representing an individual glomerulus (*LacZ*/saline, *n* = 450; *LacZ*/ADR, *n* = 300; *Tmsb4x*/ADR, *n* = 250 glomeruli; Kruskal–Wallis with Dunn post hoc test). **(f)** Representative images of glomeruli from *LacZ*/saline, *LacZ*/ADR and *Tmsb4x*/ADR treated mice immunostained to visualise synaptopodin and F-actin. White arrows indicate F-actin in synaptopodin^+^ areas. Images have been edited to crop out positive staining outside of the glomerular tuft in aid of the macro used for analysis. Quantification of **(g)** mean synaptopodin^+^ F-actin fluorescence, **(h)** area of synaptopodin^+^ covered by F-actin (µm^2^) and **(i)** percentage of synaptopodin^+^ area that was F-actin^+^ with each data point representing an individual glomerulus (*LacZ*/saline, *n* = 450; *LacZ*/ADR, *n* = 300; *Tmsb4x*/ADR, *n* = 250 glomeruli; Kruskal–Wallis with Dunn post hoc test). Scale bars = 20 µm and the white dashed line indicates glomerular tuft boundaries. Data are presented as mean ± SEM; ***P ≤ 0.001. TB4, *Tmsb4x,* thymosin β4; ADR, Adriamycin; WT1, Wilms tumour 1; *LacZ*, β-galactosidase.

Next, we assessed the effect of exogenous TB4 on podocytes. As ADR has been shown to cause podocyte loss^43^ we quantified the number of WT1^+^ glomerular podocytes^44^ (Fig. 4a). When analysing the results obtained per mouse, we did not find a statistically significant difference in podocyte number and density in the *LacZ*/ADR group compared with the *LacZ*/saline and *Tmsb4x*/ADR groups (Fig. 4b,c). Since one of the features of FSGS is that not all glomeruli are affected to the same extent^45^, we also examined podocyte number at the level of individual glomeruli by assessing a total of at least 250 glomeruli from 5 different mice in each group. This approach revealed a significant decrease in podocyte number (8.85 ± 0.31 podocytes) and density (5.31 × 10^–3^ ± 0.18 × 10^–3^ podocytes/μm^2^) in the *LacZ*/ADR group compared with *LacZ*/saline (10.94 ± 0.25 podocytes and 6.59 × 10^–3^ ± 0.13 × 10^–3^ podocytes/μm^2^ respectively) (*P* < 0.0001 in both cases). TB4 treatment prevented the ADR-induced podocyte loss and preserved podocyte number (10.38 ± 0.34 podocytes; *P* = 0.001) and podocyte density (6.57 × 10^–3^ ± 0.20 podocytes/μm^2^) (*P* < 0.0001 versus *LacZ*/ADR group) (Fig. 4d,e).

Alterations to podocyte F-actin have been associated with foot process effacement and albuminuria in vivo^10,11^ . We hypothesised that the protective effect of TB4 in ADR injury may be partly mediated by its ability to sequester G-actin and regulate F-actin polymerisation^13,14,46^ . Using synaptopodin as a podocyte marker^47^, and phalloidin to visualise F-actin filaments, we quantified F-actin in the synaptopodin-positive regions (Fig. 4f). We found that there was no difference in the mean podocyte F-actin fluorescence between any of the groups (Fig. 4g). There were also no changes to the total area (μm^2^) or percentage of podocyte area covered by F-actin, which remained at approximately 65% (Fig. 4h,i), indicating that neither ADR nor TB4 alter the amount of F-actin within podocytes.

### TB4 prevents ADR-induced podocyte F-actin reorganisation in vitro

**Figure 5:**
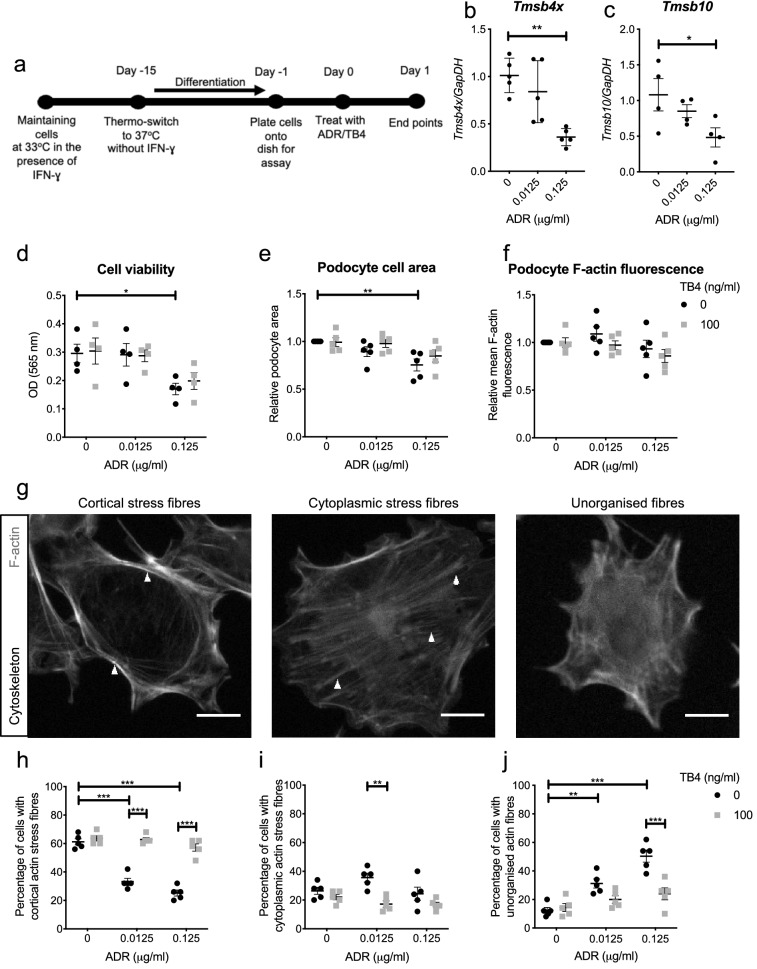
Effect of exogenous TB4 on ADR-injured podocytes in vitro. **(a)** Conditionally immortalised mouse podocytes were treated with RPMI-1640/ADR/TB4 and analysed after 24 h. Expression of podocyte *Tmsb4x* **(b)** and *Tmsb10* **(c)** mRNA after ADR/TB4 treatment (*n* = 5 independent experiments; one-way ANOVA with Tukey post hoc test). Effect of ADR/TB4 on **(d)** cell viability (*n* = 4 independent experiments; two-way ANOVA with Tukey post hoc test). Effect of ADR/TB4 on **(e)** podocyte cell area and **(f)** podocyte mean F-actin fluorescence (*n* = 5 independent experiments with 50 cells analysed per condition, per experiment; two-way ANOVA with Tukey post hoc test). **(g)** Representative images of podocytes stained with Acti-Stain™ 488 Phalloidin displaying cortical actin stress fibres, cytoplasmic stress fibres and unorganised actin fibres. Scale bar = 10 μm. Percentage of podocytes displaying **(h)** cortical actin stress fibres, **(i)** cytoplasmic stress fibres and **(j)** unorganised stress fibres (*n* = 5 independent experiments with 50 cells analysed per condition, per experiment; two-way ANOVA with Tukey post hoc test). Data are presented as mean ± SEM; *P ≤ 0.05, **P ≤ 0.01 and ***P ≤ 0.001. TB4, *Tmsb4x,* thymosin β4; *Tmsb10,* thymosin β10; ADR, Adriamycin; IFN-ɣ, interferon-gamma; OD, optical density; *GapDH,* Glyceraldehyde 3-phosphate dehydrogenase; MTT, methyltetrazolium.

Since podocyte shape and function are tightly linked to the actin cytoskeleton^7^, we next performed a detailed assessment of podocyte F-actin architecture in vitro. Cultured differentiated mouse podocytes, an established model to study the regulation of the podocyte cytoskeleton^48,49^ were treated with a low (0.0125 μg/ml) or high (0.125 μg/ml) dose of ADR and the potential of synthetic TB4 (100 ng/ml) to abrogate the effects of ADR was assessed (Fig. 5a). ADR treatment reduced *Tmsb4x* expression in cultured podocytes which was significant at the high dose (60% decrease; *P* = 0.0016; Fig. 5b) mirroring our findings in vivo. The expression of *Tmsb10* was also reduced (*P* = 0.0491) following treatment with the high dose of ADR (Fig. 5c). Treatment with 0.125 μg/ml of ADR significantly reduced podocyte viability (*P* = 0.0366) and podocyte cell area (*P* = 0.0177) compared with podocytes treated with media alone, which was not prevented by co-administration of exogenous TB4 (Fig. 5d,e). Next, we quantified podocyte F-actin. Treatment with ADR did not alter podocyte mean F-actin fluorescence, which remained unaffected by TB4 (Fig. 5f), in agreement with our in vivo finding that neither ADR nor TB4 alter the amount of F-actin within podocytes.

Finally, to study podocyte F-actin organisation in more detail, we classified F-actin arrangements into cortical actin stress fibres, cytoplasmic stress fibres or unorganised fibres (Fig. 5g). Prior to ADR administration, the majority of podocytes (61.2 ± 2.2%) had a prevalence of cortical actin stress fibres, compared with 26.4 ± 2.5% podocytes with cytoplasmic stress fibres and 12.4 ± 2.0% podocytes with unorganised actin fibres. Treatment with 0.0125 μg/ml of ADR changed this distribution with significantly reduced cortical stress fibre prevalence (33.2 ± 2.3%, *P* < 0.0001) and increased unorganised actin fibre prevalence (31.2 ± 3.2%, *P* = 0.0068) compared with untreated podocytes. Treatment with exogenous TB4 prevented the ADR-induced F-actin reorganisation and significantly increased the proportion of podocytes with cortical actin stress fibres (62.8 ± 1.4%, *P* < 0.0001) compared with the group treated with low dose ADR. Treatment with 0.125 μg/ml of ADR led to exacerbated cytoskeletal disorganisation, reducing cortical stress fibre prevalence to 25.2 ± 2.1% (*P* < 0.0001) and increasing unorganised actin fibre prevalence to 50.4 ± 4.2% (*P* < 0.0001) compared with the untreated group. Co-treatment with exogenous TB4 ameliorated the effects of ADR with cortical stress fibre frequency at 57.2 ± 2.6% (*P* < 0.0001) and unorganised actin fibre frequency at 24 ± 4.2% (*P* = 0.0001) (Fig. 5h–j).

## Discussion

In this study we have shown that ADR injury results in reduced *Tmsb4x* mRNA levels in glomeruli and particularly in podocytes. Systemic upregulation of TB4 using AAV-mediated gene therapy prevents ADR-induced albuminuria and podocyte loss in vivo and treatment with synthetic TB4 prevents ADR-induced cytoskeletal disorganisation in vitro. Thus, we have provided the first evidence that exogenous TB4 can protect the podocyte cytoskeleton and improve glomerular disease.

Previous studies have demonstrated the expression of endogenous *Tmsb4x* in mouse glomeruli predominately in podocytes^16,37,50^. The effect of glomerular disease on TB4 levels, however, is less clear. A proteomic study using the rat kidney remnant model of renal fibrosis found that TB4 levels increased threefold in sclerotic *versus* normal glomeruli^51^. Our group previously demonstrated that *Tmsb4x* levels were not altered in whole kidneys obtained from mice with glomerulonephritis or in glomerular extracts obtained from human biopsy specimens from patients with rapidly progressive glomerulonephritis or lupus nephritis^16^. These studies, however, did not assess *Tmsb4x* levels in a cell type-specific manner. Here, we have performed analysis of a scRNAseq dataset^36^ and demonstrated that ADR injury in mice results in reduced *Tmsb4x* levels specifically in podocytes. Interestingly, we also found a reduction in podocyte *Tmsb10*, suggesting that this other member of the β thymosin family may play a role in glomerular disease.

Since endogenous TB4 has a protective role in glomerular disease^16,42^, we hypothesised that treatment with exogenous TB4 would be beneficial in ADR injury. Indeed, we found that TB4 administration prevented the onset of albuminuria in mice injured with ADR. Podocyte cells are a crucial component of the glomerular filtration barrier and they are the primary target of ADR injury in the kidney^33^. We demonstrated that TB4 prevented podocyte loss in ADR-injured mice. Loss of podocytes from the glomerular tuft may result from cell death or from injury that causes podocyte detachment^2^. The actin cytoskeleton is critical to maintain podocyte shape^52^ and attachment to the GBM^8^. We developed a novel method to quantify podocyte F-actin in vivo and found that neither ADR nor TB4 affected the amount of F-actin in the podocytes. However, detailed analysis of the F-actin cytoskeleton in cultured podocytes revealed that whilst the amount of F-actin was unchanged, ADR injury induced actin disorganisation and this was prevented by treatment with TB4. These findings demonstrate that TB4 protects the podocyte cytoskeleton and prevents podocyte injury and loss which is associated with an improvement in albuminuria following ADR injury. In the epidermis, lack of endogenous TB4 results in hindered eyelid closure and hair follicle angling and defects in planar cell polarity (PCP) with impaired stability of adherens junctions, aberrant F-actin distribution and changes in cell shape^53^. PCP is also implicated in podocyte health in development and disease^54^. Van Gogh‐like 2 (Vangl2), a core PCP protein, is required for the normal differentiation of glomeruli^55,56^ and podocyte-specific deletion of *Vangl2* exacerbates experimental glomerulonephritis in mice^57,58^. It is therefore possible that some of the effects of TB4 on podocyte cells might be mediated via PCP pathways.

Previous studies have shown that endogenous and exogenous TB4 can improve inflammation in animal models of kidney injury including nephrotoxic nephritis^16^, angiotensin-II induced hypertensive nephropathy^42^ and acute ischaemia reperfusion injury^25^. In our study, we assessed the effects of TB4 in the early stages of ADR injury when macrophage infiltration is not present, demonstrating that the protective effect of TB4 is likely independent of its anti-inflammatory properties in this case.

Our study used AAV-mediated systemic gene therapy to achieve long term transgene upregulation^38^. Previous studies have used administration of TB4 protein, which maintains enhanced circulating TB4 levels for only 6 h^26^. Our strategy circumvents the quick turnover of TB4 with raised mRNA levels of *Tmsb4x* in the liver and sustained upregulation of circulating TB4 protein levels 5 weeks after AAV administration. Additionally, we demonstrate that the raised circulating TB4 levels following AAV administration can reach the podocytes. We therefore postulate that circulating TB4 can be internalised by podocytes, as previously shown in other cell types^59–61^, and modify their response to injury, however, it is also plausible that circulating TB4 may interact with extracellular receptors to modify podocyte function. Accumulation of increasing levels of TB4 protein in tissues following AAV gene therapy is likely prevented by the action of the peptidases, prolyl oligopeptidase and meprin, that hydrolyze TB4^62^ and by urinary excretion^63^. Future studies could target TB4-encoding AAVs to the kidney, however, systemic administration of AAV serotypes 1–9 has shown no efficient transduction in the kidney^38^. Transcriptional targeting^64^, synthetic AAVs^65^ and novel administration routes, such as administration by retrograde ureteral and subcapsular injections^66^ or by injection into the renal vein^67^, have shown promise and they could be utilised to achieve kidney-specific overexpression of TB4. Inducible AAVs^68,69^ would enable regulation of the timing of TB4 upregulation to assess its ability to improve the progression of established glomerular disease.

In summary, we have shown that ADR injury results in reduced levels of endogenous TB4, podocyte loss and proteinuria. Systemic gene therapy with TB4 protects the podocyte cytoskeleton and prevents proteinuria and podocyte loss. These findings suggest that treatment with TB4 could be a novel therapeutic strategy targetting the podocyte cytoskeleton to prevent podocyte injury and maintain filtration in glomerular disease.

## Methods

### scRNAseq analysis

scRNAseq analysis was performed using RStudio for Macintosh (RStudio Inc., v1.2.5042) using R (v4.0.2). The complete annotated R code used to perform the analyses and generate plots used in the manuscript has been deposited in GitHub and can be accessed at https://github.com/davidlonglab/Mason_TB4_2021.

### Data acquisition

The raw scRNAseq dataset used in this analysis was acquired from a study characterising the single-cell transcriptome of murine ADR nephropathy, nephrotoxic nephritis and diabetic kidney disease using the 10× Genomics platform^36^. Matrices of gene counts per droplet, generated after alignment of reads to genes, were acquired from the National Center for Biotechnology Information Gene Expression Omnibus (GSE146912) and are available at https://www.ncbi.nlm.nih.gov/geo/query/acc.cgi?acc=GSE146912.

### Quality control, data processing and integration

All the following analysis was performed using the Seurat toolkit^70^. The count matrices from *n* = 2 control samples (8412 cells) and *n* = 2 samples with ADR nephropathy obtained 14 days after ADR injection (8296 cells) were merged into a single object. Genes expressed in two or fewer droplets were excluded and droplets with < 200 and > 4000 detected genes and > 10% of features mapping to the mitochondrial genome were excluded. The counts were then normalized using the NormalizeData function and scaled by all detected genes using the ScaleData function before principal component analysis (PCA), using the top nine components for downstream analyses. Integration and matching of cell types between experimental conditions was performed using the Harmony package for R^71^. This process was repeated for single-cell transcriptome data derived from *n* = 2 nephrotoxic nephritis samples obtained 5 days after injection with nephrotoxic serum and *n* = 2 controls, and *n* = 2 twelve-week old *Lepr*^*Ob/Ob*^ diabetic kidney disease samples or *n* = *2* wildtype (*Lepr*^+*/*+^) controls. These datasets were analysed independently to account for differences in confounding factors such as genetic background and age between experiments and to perform individual quality control on each dataset. For the nephrotoxic nephritis dataset, droplets with 200–7500 genes were included whereas droplets with 200–6000 genes were included from the *Lepr*^*Ob/Ob*^ dataset. For both datasets, droplets with > 10% mitochondrial features were excluded.

### Clustering, cell type identification and counting

Shared nearest neighbor graphing was performed using the FindNeighbors function. Unsupervised clustering was performed with the FindClusters function using the Louvain algorithm and a resolution of 0.4, generating 14 transcriptionally distinct clusters, before dimension reduction using Uniform Manifold Approximation and Projection (UMAP). Cell type identification was performed by assessing the top ten differentially expressed genes per cluster calculated using the FindAllMarkers function and canonical markers for glomerular cell types were compared from previous scRNAseq studies^36,72,73^. By grouping clusters with a common cell identity together, ten glomerular cell types were subsequently identified and assigned. The number of cell types by experimental condition was exported and graphed in Prism (GraphPad, v9.0.0).

### Comparison of *Tmsb4x* or *Tmsb10* expression

The FindAllMarkers function was used to compare the scaled expression of *Tmsb4x* or *Tmsb10* between disease and control datasets. The average log fold change was calculated for podocytes or all cell types within the glomerular tuft (glomerular endothelial cells, mesangial cells and podocytes) between experimental conditions. Wilcoxon Rank Sum tests was used to assess statistical significance, with an adjusted *P* value of ≤ 0.05. Expression values were exported into Prism and graphed using violin plots.

### Adeno-associated viral generation

The recombinant AAV-*Tmsb4x* and AAV-*LacZ* vectors were produced using triple transfection in HEK293 cells. Cells were harvested and virus was purified by iodixanol-gradient centrifugation. The virus was further purified using Sepharose G100 SF resin (Merck, Darmstadt, Germany) in Econopac colums (Bio-Rad, Watford, UK). Virus was concentrated in PBS using Amicon Ultra-15 Centrifugal Filter Units (Merck) and stored at 4 °C^31,74,75^. Viral titre was quantified by inverted terminal repeat probe quantitative polymerase chain reaction (PCR). Helper plasmid delta F6 was purchased from Puresyn (Malvern, PA).

### Experimental animals and procedures

All experiments were carried out according to a UK Home Office project in accordance with the UK Animals (Scientific Procedures) Act 1986 and the ARRIVE guidelines and with institutional ethical approval (University College London Local Ethics Committee). Male BALB/c mice^76^ aged between 7–10 weeks were administered with AAV (sub-serotype 2/7; 5 × 10^12^ viral particles per mouse) expressing *LacZ* (AAV-*LacZ*) or *Tmsb4x* (AAV*-Tmsb4x*) via the tail vein. Male C57BL/6 *Tmsb4x* knockout mice^76^ were administered with 2 × 10^12^ AAV-*LacZ* or AAV*-Tmsb4x* viral particles. To induce glomerular injury, mice were intravenously injected with 10 mg/kg of ADR (Merck), a chemotherapeutic drug that has toxic effects on podocytes and replicates some of the features of human focal segmental glomerulosclerosis (FSGS)^33^ or vehicle (0.9% saline) 21 days after AAV administration.

### Renal function

Urine was collected from mice by housing them individually in metabolic cages overnight. Blood samples were collected from the lateral saphenous vein. Albumin concentrations were measured by enzyme-linked immunosorbent assay^77,78^ (Bethyl Laboratories, Montgomery, TX). A commercially available kit was used to measure BUN (BioAssay Systems, Hayward, CA)^79^ .

### TB4 enzyme linked immunosorbent assay (ELISA)

The plasma concentration of TB4 was determined by ELISA based on the protocol previously described by Mora et al.^26^. Standards of 10,000, 5000, 2500, 1250, 625, 312.5, 156, 78 and 39 ng/ml TB4 were prepared using synthetic TB4 (ReGeneRx Biopharmaceuticals Inc, Rockville, MD) diluted in incubation buffer [(pH 7.4, Na_2_HPO_4_ (0.01 M), NaCl (0.15 M), Tween-20 (0.055% v/v), BSA (1% v/v)]. Equal volumes of standards or samples, incubation buffer and TB4 antibody prediluted at 1:4000 (AF6796; R&D Systems, Minneapolis, MN) were added to sterile borosilicate tubes and incubated overnight at 4 °C. A flat bottom 96 well plate was coated with 100 μl of 50 ng/ml recombinant TB4 in carbonate-bicarbonate buffer and incubated overnight at 4 °C. Negative control wells were coated with buffer only. The plate was washed with washing buffer [(pH 7.4, Na_2_HPO_4_ (0.01 M), NaCl (0.15 M), CaCl_2_ (1 mM), MgCl_2_ (0.5 mM), Tween-20 (0.55% v/v)], blocked with 200 μl of blocking buffer (5% dry fat milk in incubation buffer) and 100 μl of each standard and sample were added to the appropriate wells and incubated for 2 h. Following washing, 100 μl of goat anti-sheep HRP-conjugated secondary antibody (61–8620, Thermo Fisher Scientific) diluted 1:2000 in incubation buffer was added to each well and incubated for 1 h before washing. Substrate solution (100 μl per well) containing equal parts stabilised H_2_O_2_ and stabilised tetramethylbenzidine was added and 15 min later the reaction was stopped with the addition of 50 μl of 2 M sulphuric acid per well and absorbance was read at 450 nm using a plate reader (M200 Pro, Tecan, Männedorf, Switzerland).

### Tissue processing and immunostaining

Tissues were fixed in 4% paraformaldehyde in PBS. Wax Sections (5 μm thick) were prepared following tissue dehydration and paraffin embedding. To prepare cryosections (8 μm thick), tissues were placed overnight in 30% sucrose in PBS and embedded in Tissue-Tek optimal cutting temperature compound (Agar Scientific, Stansted, UK).

Glomerular morphology was examined by two blinded assessors and designated as normal (little PAS‐positive material and normal capillary loops) or abnormal (PAS-positive material in > 25% of the glomerular tuft). Fifty glomeruli were assessed per mouse and results for each category were expressed as a percentage of the total glomeruli assessed.

Immunohistochemistry was performed for TB4 (AF6796, R&D Systems) followed by secondary rabbit anti-sheep antibodies (Thermo Fisher Scientific, Waltham, MA) and ImmPRESS polymer anti-rabbit IgG reagent (Vector Laboratories, Burlingame, CA) conjugated to horseradish peroxidase and detected by 3,3′-diaminobenzidine. Images were obtained on a Leica DM5500 B brightfield microscope (Leica Biosystems, Milton Keynes, UK).

Immunofluorescence was performed^80^ using primary antibodies against Wilms Tumour 1 (WT1) (AB89901, Abcam, Cambridge, UK), synaptopodin (163-004-SY, Synaptic Systems, Goettingen, Germany), TB4 (AF6796, R&D Systems) and F4/80 (MCA497R, Bio-Rad), followed by appropriate AlexaFluor594 and AlexaFluor488 (Thermo Fisher Scientific) secondary antibodies. Negative controls consisted of omission of primary antibodies. Acti-Stain 488™ Phalloidin (Cytoskeleton, Denver, CO) was used to visualise actin filaments. Images were acquired using a Zeiss Laser Scanning 880 confocal microscope with a 63 × NA1.4 Oil Plan Apochromat objective (Carl Zeiss, Oberkochen, Germany).

The number of podocytes (WT1^+^) within the glomerular tuft and podocyte density (WT1^+^ cells/glomerular area measured using ImageJ^81^) were quantified. F4/80^+^ cells within the glomerular tuft and in the peri-glomerular area were counted. A macro was generated (https://github.com/DaleMoulding/Fiji-Macros/blob/master/README.md#podocyte-f-actin--synaptopodin) for automated quantification of podocyte F-actin. A Gaussian blur with a Sigma (radius) value of 2.0 was applied to each channel to create a solid mask of synaptopodin outlining the podocyte area. The mean fluorescence of F-actin within the synaptopodin positive area was measured along with the total area (μm^2^) and percentage area of F-actin in the synaptopodin positive regions. All measurements were performed in 50 glomeruli per mouse.

### Cell culture

Mouse podocytes^48^ were cultured as described^56^ and allowed to differentiate for 14 days. Cells were treated with 100 ng/ml synthetic TB4^82^ (ReGeneRx Biopharmaceuticals Inc) and either a low (0.0125 μg/ml) or high (0.125 μg/ml) dose of ADR for 24 h.

Cell viability was determined by the methyltetrazolium assay. To visualize F-actin filaments, podocytes were fixed in 4% paraformaldehyde and 4% sucrose and stained with Acti-stain™ 488 Phalloidin and 50 cells per condition were assessed. The area of each cell and the mean F-actin fluorescence were quantified using ImageJ. Actin filaments were classified as cortical stress fibers, which are found in the periphery of the cell, cytoplasmic stress fibers, which transverse the cell body, or unorganised actin lacking any specific arrangement. Each cell was scored depending on the prevalent arrangement observed.

### Quantitative real-time PCR (qRT-PCR)

RNA extracted from mouse whole-kidney (500 ng), or cultured podocytes (100 ng) was used to prepare cDNA (iScript kit, Bio-Rad), and qRT-PCR was performed as described previously^78^ with *GapDH* as a housekeeping gene. All measurements were performed in duplicate.

### Statistical analysis

All samples were assessed by independent observers blinded to treatment group. Data are presented as mean ± SD and were analysed using GraphPad Prism v9 (GraphPad Software, La Jolla, CA). Normal distribution was assessed by Shapiro–Wilk test. For comparisons of two groups, data were analysed using a t test. When three or more groups were assessed, one-way ANOVA with Tukey multiple comparison post hoc tests was used. Data affected by two variables were analysed using two-way ANOVA with Tukey multiple comparison post hoc tests. For analysis of individual glomeruli, data were analysed by Kruskal–Wallis non-parametric test followed by Dunn post hoc tests. Statistical significance was accepted at *P* ≤ 0.05.

## Supplementary Information

Supplementary Figures.

## Data Availability

The datasets generated during and/or analysed during the current study are available from the corresponding author on reasonable request.
