# Supplementary material for: Systemic gene therapy with thymosin β4 alleviates glomerular injury in mice
Source: Sci Rep. 2022 Jul 16;12:12172. doi: 10.1038/s41598-022-16287-z (PMC9288454; doi:10.1038/s41598-022-16287-z)
Supplement: Supplementary file 1 — Supplementary Figures. [file 41598_2022_16287_MOESM1_ESM.pdf]

Supplementary Data

Supplementary Figure 1

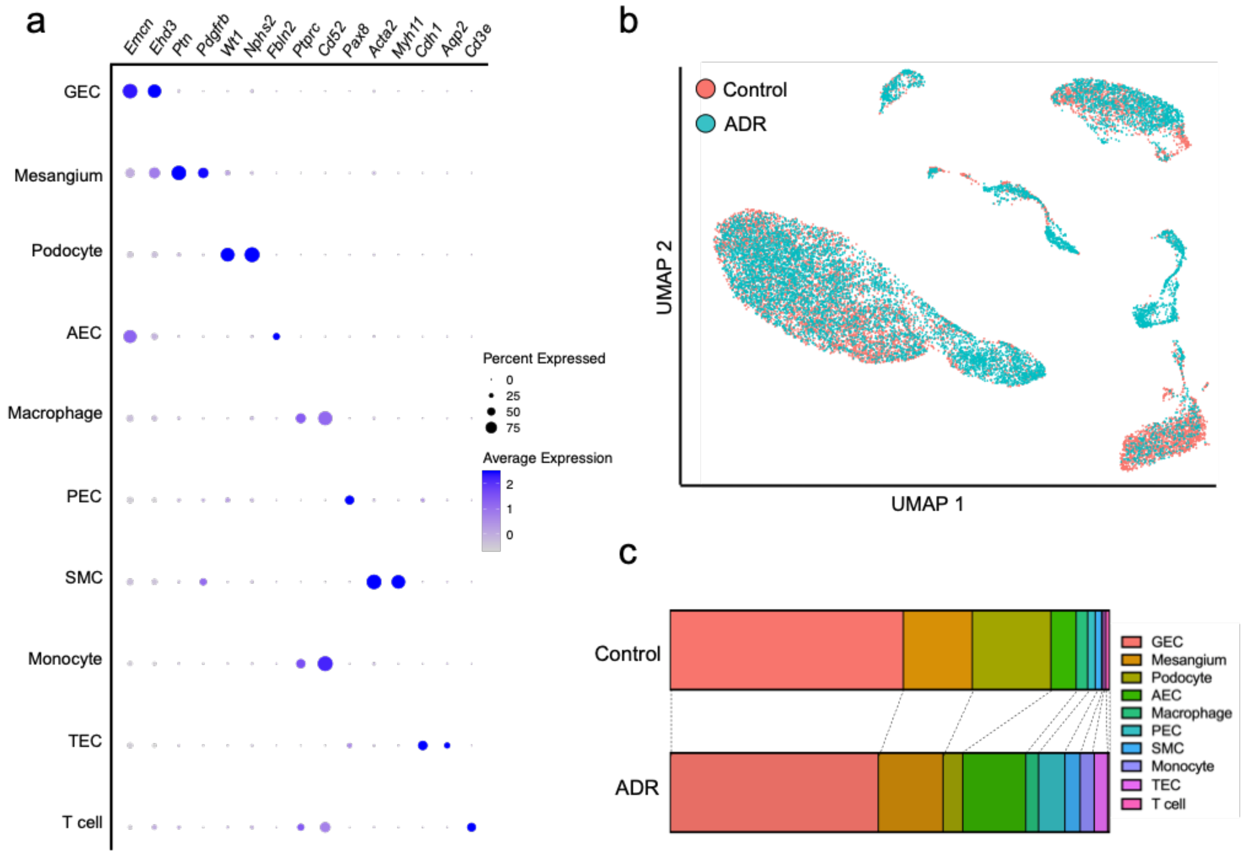

Supplementary Figure 1. Cell type identification and comparison of cell type proportions within single-cell RNA sequencing data. (a) Dotplot showing enrichment of canonical markers between cell types within the single-cell RNA sequencing (scRNAseq) dataset. The markers include endomucin (*Emcn)* and Eps15 homology domain-containing protein 3 *(Edh3)* for glomerular endothelial cells (GEC), pleiotrophin *(Ptn)* and platelet-derived growth factor receptor beta *(Pdgfrb)* for mesangial cells, Wilms' tumour 1 *(Wt1)* and podocin (*Nphs2)* for podocytes, *Emcn* and fibulin 2 *(Fbln2)* for arterial endothelial cells (AEC), protein tyrosine phosphatase receptor type C *(Ptprc)* and campath 1 antigen *(Cd52)* for monocytes and macrophages, paired box gene 8 *(Pax8)* for parietal epithelial cells (PEC), actin alpha 2 (*Acta2*) and myosin heavy chain 11 (*Myh11*) for

smooth muscle cells (SMC), E-cadherin (*Cdh1*) and aquaporin 2 (*Aqp2*) for tubular epithelial cells (TEC) and T cell surface glycoprotein CD3 epsilon chain (*Cd3e*) for T cells. **(b)** Uniform manifold approximation and projection (UMAP) grouped by experimental condition in the scRNAseq dataset. The UMAP corresponds to **Figure 1a**, showing concordance of cell types between Adriamycin nephropathy (ADR) and control. **(c)** Bar graphs comparing the proportions of cell types between ADR and control. In the control dataset, *n* = 4,402 GECs, *n* = 1,302 mesangial cells, *n* = 1,486 podocytes, *n* = 477 AECs, n = 218 macrophages, *n* = 143 PECs, *n* = 118 SMCs, *n* = 34 monocytes, *n* = 47 TECs and *n* = 69 T cells were detected. In the ADR dataset *n* = 3,895 GECs, *n* = 1,239 mesangial cells, *n* = 378 podocytes, *n* = 1,207 AECs, *n* = 245 macrophages, *n* = 505 PECs, *n* = 290 SMCs, *n* = 271 monocytes, *n* = 256 TECs and *n* = 36 T cells were detected.

Supplementary Figure 2

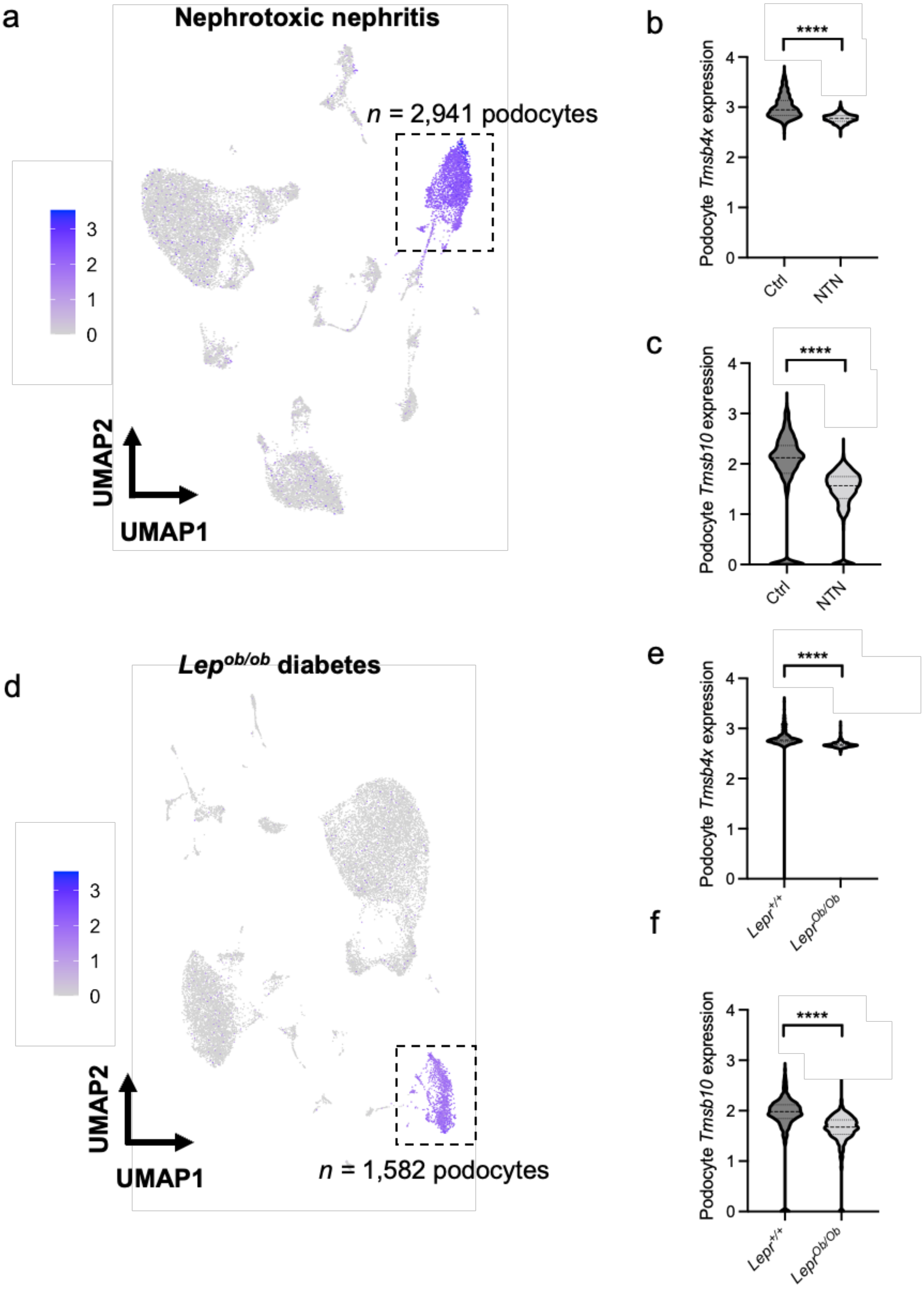

Supplementary Figure 2. Reduction in podocyte *Tmsb4x* and *Tmsb10* mRNA across multiple murine models of glomerular disease. (a) UMAP of scRNAseq data from two control mice and two mice with nephrotoxic nephritis (NTN). Expression of nephrin (*Nphs1)* was used to identify a cluster of podocytes from the aggregated dataset. Violin plots comparing the podocyte expression of *Tmsb4x* **(b)** and *Tmsb10* **(c)** between experimental conditions. In NTN podocytes (n = 1,491) compared to control (n = 1,450), average log fold decreases of 0.36 and 0.84 were detected for *Tmsb4x* and *Tmsb10* respectively (****: adjusted P values < 0.0001). **(d)** UMAP of scRNAseq data from two wildtype ($Lepr^{+/+}$) and two $Lepr^{Ob/Ob}$ diabetic mice. Expression of nephrin (*Nphs1)* was used to identify a cluster of podocytes from the aggregated dataset. Violin plots comparing the podocyte expression of *Tmsb4x* **(e)** and *Tmsb10* **(f)** between experimental conditions. In $Lepr^{Ob/Ob}$ podocytes (n = 869) compared to wildtype control (n = 713), average log fold decreases of 0.20 and 0.45 were detected for *Tmsb4x* and *Tmsb10* respectively (*: adjusted P values < 0.0001).

Supplementary Figure 3

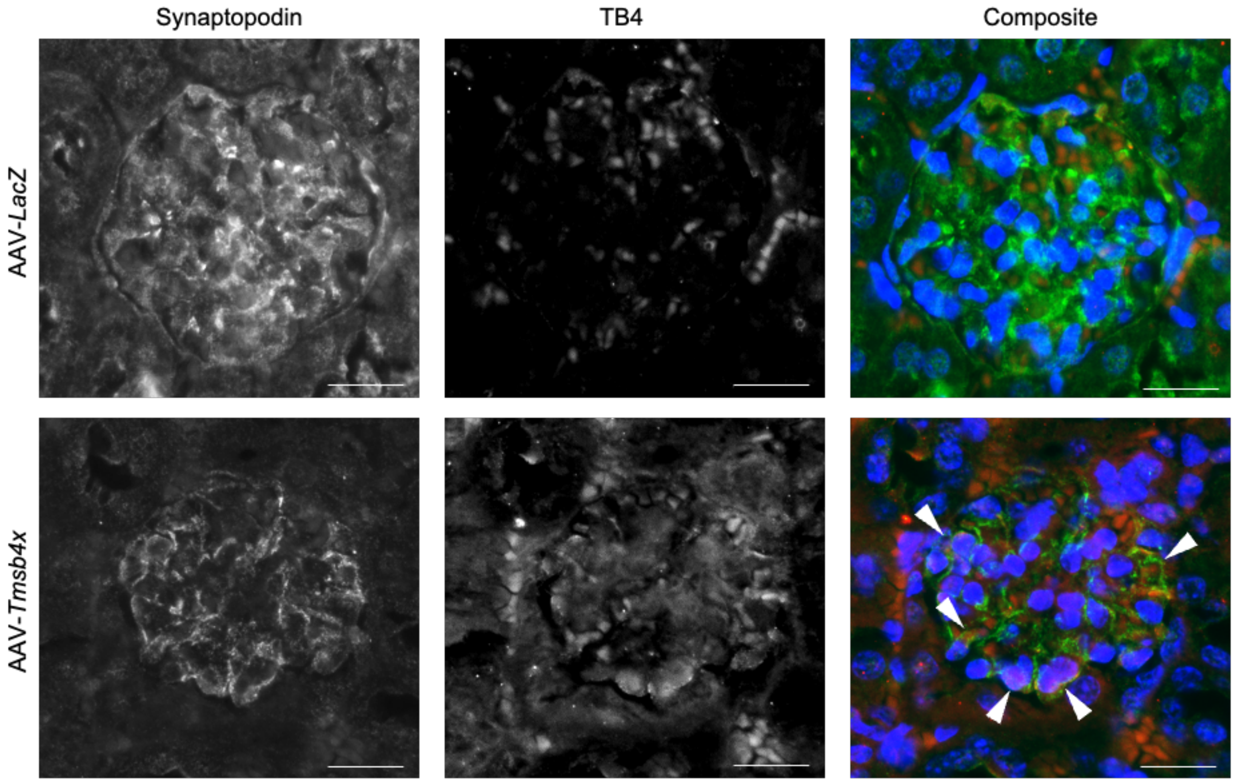

Summplementary Figure 3. AAV-derived thymosin β4 can reach the podocyte cells *in vivo.*

Representative images of staining for TB4 peptide in glomeruli of TB4 knockout mice injected with AAV-*Tmsb4x* or AAV-*LacZ*. Podocyte cells are identified by staining with synaptopodin. White arrowheads indicate the presence of TB4 peptide in podocyte cells in AAV-*Tmsb4x* injected mice. Scale bars = 20 µm. TB4, *Tmsb4x*, thymosin β4; *LacZ*, β-Galactosidase.

Supplementary Figure 4

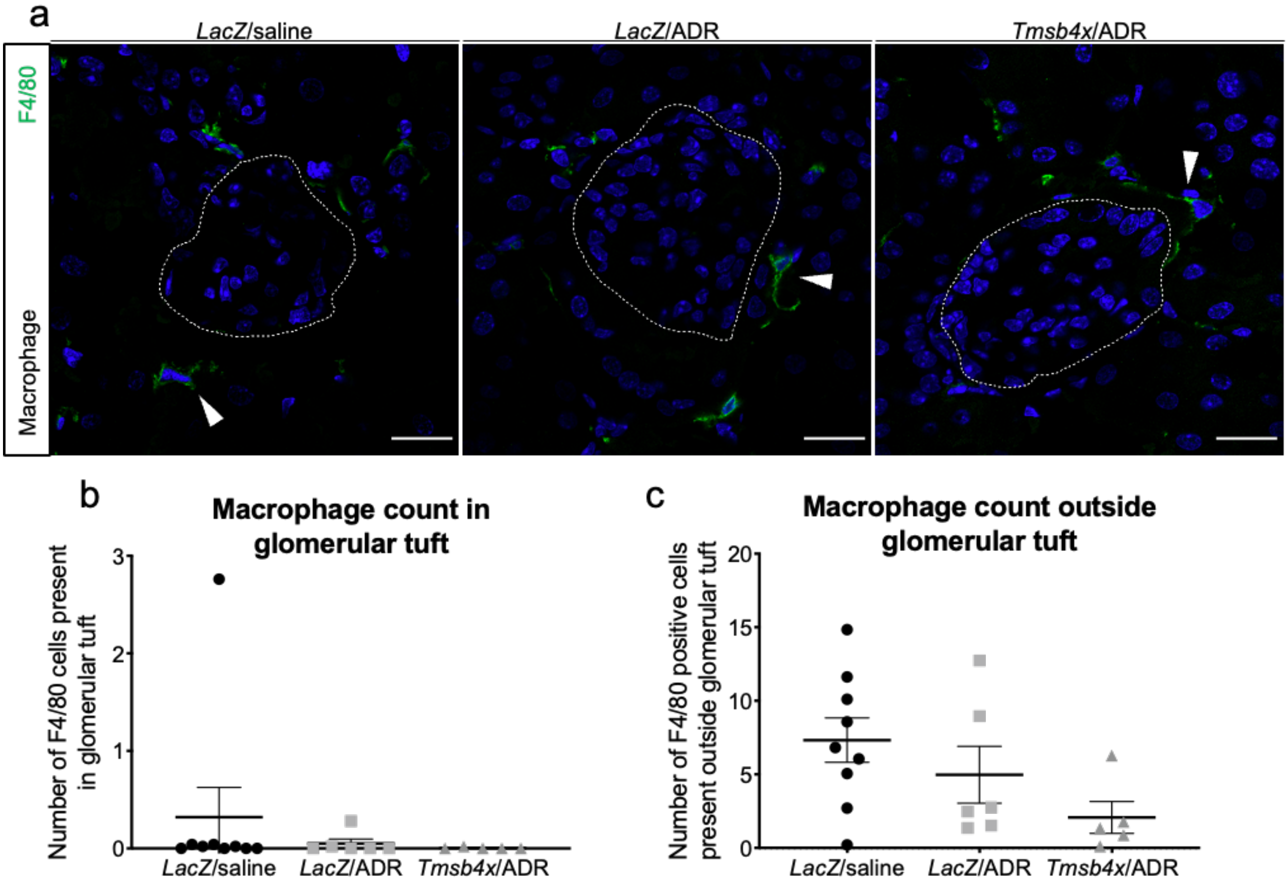

Supplementary Figure 4. Adriamycin does not cause macrophage infiltration at 14 days. (a)

Representative images of F4/80 expression around *LacZ*/saline, *LacZ*/ADR and *Tmsb4x*/ADR glomeruli. White arrowheads indicate positive F4/80 staining. White dashed line indicates the glomerular tuft. Quantification of F4/80 positive cells **(b)** inside the glomerular tuft and **(c)** outside the glomerular tuft. Individual data points represent average values per mouse (*LacZ*/saline, *n* = 9; *LacZ*/ADR, *n* = 6; *Tmsb4x*/ADR, *n* = 5 mice; one-way ANOVA with Tukey *post hoc* test) and 50 glomeruli were assessed per mouse. Data are presented as mean±SEM. Scale bars = 20 µm. TB4, *Tmsb4x*, thymosin β4; ADR, Adriamycin; *LacZ*, β-Galactosidase.
